# Salmonella enterica Newserovar Abeokuta Genome Sequence, Strain OG19FER4 Isolated from Poultry Feed in Nigeria

**DOI:** 10.1128/mra.00489-22

**Published:** 2022-09-28

**Authors:** Idowu O. Fagbamila, Alejandra Hernandez-Segura, Maaike van den Beld, Kirsten Mooijman, Massimiliano Orsini, Olawunmi T. Ajayi, Sati Ngulukun, Alexander Ray Jambalang, Nancy Sati, Paulinus Emennaa, Paul I. Ankeli, Maryam Muhammad, Lisa Barco

**Affiliations:** a Bacterial Research Division, National Veterinary Research Institute, Vom, Plateau State, Nigeria; b Centre for Infectious Disease Control, National Institute for Public Health and the Environment (RIVM), Bilthoven, the Netherlands; c European Union Reference Laboratory (EURL) for Salmonella, National Institute for Public Health and the Environment (RIVM), Bilthoven, the Netherlands; d OIE and National Reference Laboratory for Salmonellosis, Istituto Zooprofilattico Sperimentale delle Venezie, Legnaro (PD), Italy; University of Maryland School of Medicine

## Abstract

This report announces the genome of a newly confirmed Salmonella serovar (Salmonella enterica serovar Abeokuta) that was isolated from a poultry feed sample collected on a farm in Abeokuta, capital of Ogun State in Nigeria. Salmonella Abeokuta has not been identified outside Nigeria, nor does it appear to be a cause for concern for animal and human health.

## ANNOUNCEMENT

Salmonella is a common cause of gastroenteritis in humans (1) and even life-threatening disease (2). Moreover, Salmonella can lead to economic losses (3, 4).

Salmonella surveillance was conducted on poultry farms in Nigeria (2012 to 2015) (5). Here, we report the genome sequence of one inconclusive serovar that was isolated from a poultry feed sample collected on 13 February 2012 (latitude, 7.13N; longitude, 3.27E) in Abeokuta (Ogun State, Nigeria). The strain was isolated according to ISO 6579:2002 (6) and confirmed by ISO/TR 6579-3:2014 (7) as Salmonella enterica subsp. *enterica* 30:d:z_6_. This seroformula had never been published in the White-Kauffmann-Le Minor scheme (8), and the World Health Organization (WHO) Reference Center of Salmonella confirmed a new serovar by phenotypic and genotypic typing, now called Salmonella enterica subsp. *enterica* serovar Abeokuta (*S*. Abeokuta).

The isolate was cultured in tryptic soy broth (Sigma-Aldrich, Inc.) at 37°C overnight, and genomic DNA was extracted using the GenElute bacterial genomic DNA kit (Sigma-Aldrich, Inc.) according to the manufacturer’s instructions. Whole-genome sequence was obtained using short-read and long-read sequencing approaches. Short-read libraries were prepared using the Illumina Nextera XT DNA library prep kit (Illumina, Inc.) and sequenced using Illumina NextSeq 500/550, resulting in 5,253,397 paired-end reads (150 bp), with a theoretical sequencing depth of about 150×. For long-read sequencing, the library was prepared with a rapid barcoding kit (SQK-RBK004) (no DNA shearing or size selection), loaded onto an R9.4.1 flow cell (FLO-MIN106), and sequenced in a GridION platform for 48 h. Live base calling was performed using GridION software (MinKNOW v21.05.25, MinKNOW GUI v4.0.20, and Ont-kingfisher-ui-gridion v4.3.28) obtaining 9,676 good-quality long reads with an average length of 8,591.66 bp, providing an average sequencing depth of 16×. Filtering was done using FastP (v0.20.1) (9) for short reads, and default filtering was performed with the minKNOW software followed by NanoFilt 2.8.0 (10) and Filtlong v0.2.1 (https://github.com/rrwick/Filtlong) for long reads. The quality control used FastQC (v0.11.9) (https://www.bioinformatics.babraham.ac.uk/projects/fastqc/) for short reads and PycoQC 2.5.2 for long reads, resulting in an *N*_50_ read length of 20,549. The seroformula was confirmed using SeqSero2 (11). A hybrid assembly was performed using Unicycler (v0.4.8) (12), including a rotation step to ensure the *dnaA* gene was at the start of the forward strand. This resulted in one circular contig of 4,957,438 bp in length and 52.01% GC content. Analysis with CheckM (v1.1.3) (13) determined 99.54% genome completeness. The genome was annotated using PGAP (6.0) (14), which returned 4,788 genes: 4,564 protein-coding genes, 83 tRNAs, and 7 complete rRNA operons. ResFinder 4.0 software (15) predicted resistance to nalidixic acid, ciprofloxacin, amikacin, and tobramycin. The strain 10734/15 genome sequence was submitted to Enterobase (16) by the WHO Reference Center of Salmonella, resulting in 7-locus multilocus sequence type (MLST) type 8600, a unique ST. Further, using Salmonella cgMLST V2 plus HierCC V1 (17), the genome belongs to superlineage HC2000/219927, which includes Salmonella enterica serovar Kakikoka only.

### Data availability.

Both long and short reads were submitted to the SRA database under the BioProject accession identifier (ID) PRJNA816352, with the accession IDs SRR18503845 and SRR18503846, respectively. The annotated genome sequence is available in the NCBI RefSeq database (accession ID NZ_CP093445.1).
